# My E-learning Experience as a Medical Student during the COVID-19 Pandemic

**DOI:** 10.31729/jnma.5124

**Published:** 2020-08-31

**Authors:** Pearlbiga Karki

**Affiliations:** 1 Nepalese Army Institute of Health Sciences, Sanobharyang, Kathmandu, Nepal

The COVID-19 pandemic has disrupted the lives of people worldwide in various ways. Globally, as of 30 August 2020, there have been 21,294,845 confirmed cases of COVID-19, including 761,779 deaths.^1^ Along with the alarming number of cases and deaths, economic and social crises, we can also notice the frail education system. The Government of Nepal had requested social distancing and implemented lockdown. Therefore, along with all other educational institutions, my medical college was also closed. The ministry of education of Nepal postponed all the exams of the country as well. It included the third-year medical board exams, for which I had eagerly awaited.

After my lockdown began, I spent the start of it engaging in hobbies that I had left behind during my childhood. However, I still had so much more leisure time on my hands. Being recently buried in textbooks, I did not seek them again. That is why when my medical institution started E-learning from the 36th day of lockdown, I was excited. Since I was a student who mostly confined to textbooks and lecture notes, along with the excitement, came many challenges. The journey of my E-learning began with the Zoom app. We eventually switched to Google Meet and Google classroom. These apps offer access to a large number of high-quality, peer-reviewed, sharable e-learning materials.^2^ Initially, technical errors like logging in and sharing the presentations took half of the time allocated to us for our online lectures. Nevertheless, after two to three classes, the teachers and we were familiar with the apps.

At first, the E-learning consisted mostly of the fifth-year subjects we studied during our Third-year of MBBS. We were already well adapted to the teachers and their teaching techniques.

After a fortnight, our medical institution decided that even though we did not give our third-year board exams, we would still get the opportunity to study fourth-year subjects. The teachers of these subjects, however, were new to us. It took quite a while for us to understand how they taught and how they expected us to behave during the online classes. It also did not help that along with the majority of the fourth-year subjects, additional theory classes of the fifth year subjects were all taught to us online. It took me by surprise when I looked in the Google classroom and found total subjects taught in the online classes of the fourth-year to be fourteen.

## STRENGTHS OF E-LEARNING

The overload of information found online and given by our teachers in the online classes made me anxious. However, with the help of online group discussions with my friends and with the right management of time, I finally found the solution. I realized that these helpful group discussions were encouraged due to E-learning. In these group discussions, we divided a topic into various parts and allocated one to each. We then made presentations using pictures and videos and discussed them using Google Meet. E-learning made the implementation of many instructional methods easier.^3^

Slowly, I caught up with the ongoing topics and understood my teachers better. I found out that E-learning improves student-teacher relationships. Via Internet-based learning, we were able to contact the academic staff online outside the scheduled contact hours.^4^ It is also helpful to teachers to update the materials that they provide us online compared to printed materials used in traditional teaching methods.^2^ E-learning technologies offer learners control over content, learning sequence, pace of learning, time, and often media, allowing them to tailor their experiences to meet their personal learning objectives. In diverse medical education contexts, e-learning appears to be at least as effective as traditional instructor-led methods such as lectures. Students do not see e-learning as replacing traditional instructor-led training but as a complement to it, forming part of a blended-learning strategy. Innovations in e-learning technologies point toward a revolution in education, allowing learning to be individualized (adaptive learning

E-learning is a blessing to all of us, who have returned to our home town due to the COVID-19 pandemic. It facilitates the teaching of students scattered across different places in the same city, different cities, and even different countries. The result of this is that we got the opportunity to participate in the same educational activities regardless of physical location.^5^ The environment for e-learning also encourages us to depend on ourselves for the reason that teachers are no longer the solitary source of knowledge.^6^

## WEAKNESSES OF E-LEARNING

Despite its strengths, E-learning also has many limitations. Bedside learning we learn in the hospital make up an integral part of the fourth-year curriculum of MBBS. It is the year we mainly focus on communication with the patients and learn from them. Teaching communication skills requires real group meetings and face-to-face interaction. In this aspect, E-Learning may not benefit every field of medicine and allied health professional education.^4^ E-learning with no actual patient interaction will eventually cause anxiety among the students while facing one. They will also be less empathetic towards the patients since attitudes of doctors and communication skills are closely linked. They ultimately influence the quality of medical care.^7^ Medicine of the present world demands a high level of competency in both clinical examination and performing a procedure in patients.^8^ E-learning alone will not help the students develop effective practical and clinical skills.^6^ For example: Watching a tutorial about cranial nerve examination is not as effective as a cranial nerve examination on a real patient.

To access E-learning materials, we require a PC or a smart device (tablet or phone), as well as access to the Internet.^4^ In Nepal, only 21.4 percent of the population is online compared to 77 percent in other developed countries.^4,9^ Therefore, E-learning may not be possible for every student. Even when possible, many technical errors are still present, for example, slow internet connection and the absence of electricity from time to time. The process of effective E-learning can be quite expensive too.^5^economies of scale, and novel instructional methods, while disadvantages include social isolation, up-front costs, and technical problems. Web-based learning is purported to facilitate individualised instruction, but this is currently more vision than reality. More importantly, many WBL instructional designs fail to incorporate principles of effective learning, and WBL is often used for the wrong reasons (eg for the sake of technology In a country where most households do not have a WiFi connection, students should buy data packages for E-learning. The economic burden due to lockdown and closed offices as a consequence of the COVID-19 pandemic is also huge. Hence, it adds to the decreasing economy of many students and their families and increases their extra expenses.

The E-learning process is new to all of us. The sudden change from a classroom filled with friends and teachers to only one person behind a screen can be quite a sad experience. Over time, it may lead to a perception of social isolation.^5^ During the COVID-19 pandemic, different mental problems are emerging, both recurrent and new ones, mainly including mood and anxiety disorders.^10^ This perception of social isolation will add up to it and affect the students.

The other limitation of E-learning that I came to know about was that we are easily distracted during our online lectures. Even minor inconvenience leads to decreasing satisfaction, and course participation.^3^ Because the students are at home due to the COVID-19 pandemic, there's a lot of potential for distractions: Parents asking the students to do household chores or requesting their help on technical errors of their online meetings.

## HOW CAN WE IMPROVE IT?

The limitations, however, should not discourage the process of E-learning. Instead, they should provide a way for improvements. With the continuous advancement of technology and digitalization of everything, we need to focus on the factors that make E-learning easier. For this, we should carefully consider situational factors regarding it.^11^ They include the availability of reliable technological resources, learner satisfaction, cost-effectiveness, detailed planning, and regular feedback. We should encourage the evaluation of the effectiveness of E-learning along with student inputs before implementing it. We should also focus on active student participation and motivate discipline and concentration during the online classes. We should equip ourselves with strategies that help us self-regulate our learning, especially during online learning programs.^12^ This being highly important when learning is acquired through online learning programs. Nonetheless, such research has been scarce with Vocational Education and Training (VET).

Though all these factors are for effective E-learning, in the context of Nepal, we need to first focus on factors that make E-learning possible. They include the availability of reliable technological resources, detailed planning, and cost-effectiveness.

## CONCLUSIONS

E-Learning, like any other form of learning, is personal. How we utilize the benefits of it and find solutions to its limitations depend on us. It is incumbent upon each one of us to take it as an opportunity and be flexible in this period of global crisis. After the end of the COVID-19 pandemic, when the traditional teaching method will inevitably resume, we should incorporate E-learning along with it- for a better and effective education system. We, the medical students, should learn how to take the best of both worlds to become both empathetic and competent doctors.

## Conflict of Interest

**None.**
